# Using the TAP Component of the Antigen-Processing Machinery as a Molecular Adjuvant

**DOI:** 10.1371/journal.ppat.0010036

**Published:** 2005-12-30

**Authors:** Timothy Z Vitalis, Qian-Jin Zhang, Judie Alimonti, Susan S Chen, Genc Basha, Alex Moise, Jacqueline Tiong, Mei Mei Tian, Kyung Bok Choi, Douglas Waterfield, Andy Jeffries, Wilfred A Jefferies

**Affiliations:** 1 The Michael Smith Laboratories, University of British Columbia, Vancouver, British Columbia, Canada; 2 Biomedical Research Centre, University of British Columbia, Vancouver, British Columbia, Canada; 3 Department of Microbiology and Immunology, University of British Columbia, Vancouver, British Columbia, Canada; 4 Department of Zoology, University of British Columbia, Vancouver, British Columbia, Canada; 5 Department of Oral Biology, Faculty of Dentistry, University of British Columbia, Vancouver, British Columbia, Canada; 6 Department of Medical Genetics, University of British Columbia, Vancouver, British Columbia, Canada; Scripps Research Institute, United States of America

## Abstract

We hypothesize that over-expression of transporters associated with antigen processing (TAP1 and TAP2), components of the major histocompatibility complex (MHC) class I antigen-processing pathway, enhances antigen-specific cytotoxic activity in response to viral infection. An expression system using recombinant vaccinia virus (VV) was used to over-express human TAP1 and TAP2 (VV-hTAP1,2) in normal mice. Mice coinfected with either vesicular stomatitis virus plus VV-hTAP1,2 or Sendai virus plus VV-hTAP1,2 increased cytotoxic lymphocyte (CTL) activity by at least 4-fold when compared to coinfections with a control vector, VV encoding the plasmid PJS-5. Coinfections with VV-hTAP1,2 increased virus-specific CTL precursors compared to control infections without VV-hTAP1,2. In an animal model of lethal viral challenge after vaccination, VV-hTAP1,2 provided protection against a lethal challenge of VV at doses 100-fold lower than control vector alone. Mechanistically, the total MHC class I antigen surface expression and the cross-presentation mechanism in spleen-derived dendritic cells was augmented by over-expression of TAP. Furthermore, VV-hTAP1,2 increases splenic TAP transport activity and endogenous antigen processing, thus rendering infected targets more susceptible to CTL recognition and subsequent killing. This is the first demonstration that over-expression of a component of the antigen-processing machinery increases endogenous antigen presentation and dendritic cell cross-presentation of exogenous antigens and may provide a novel and general approach for increasing immune responses against pathogens at low doses of vaccine inocula.

## Synopsis

The development of protective vaccines against infectious diseases such as AIDS, SARS, and West Nile virus has become a societal priority but remains a scientific challenge. In recent years, the threat of bioterrorism agents such as anthrax and smallpox has heightened the need for the rapid development of effective new vaccines. One of the major stumbling blocks to the implementation of any vaccine is the toxic side effects on the vaccine candidate. For example, a significant number of doses of a new vaccine against smallpox have been commissioned, but approximately 20% of the individuals targeted to be inoculated will suffer toxicity due to vaccination. Furthermore, an additional difficulty in the production of vaccines is the creation of sufficient doses to vaccinate a large population. The authors have identified a novel approach that appears to address these issues. They demonstrate that the inclusion, in low doses of vaccines, of a normal component of the antigen-processing pathway, the transporter associated with antigen processing (TAP), confers protective immunity against lethal viral loads during viral challenges. This new paradigm is shown to be applicable to many viruses, including poxviruses, and could significantly advance the creation of new vaccines and improve those that already exist.

## Introduction

Major histocompatibility complex (MHC) class I molecules are highly polymorphic cell-surface glycoproteins, which function to bind peptides for presentation to cytotoxic lymphocytes (CTLs) following microbial infection or cell transformation [1–3]. In humans, a number of genes located mainly in the MHC class II region of Chromosome 6 are responsible for the generation, assembly, and transport of MHC class I molecules, referred to as the antigen-processing pathway. These genes include (1) the proteasome components, low molecular-weight polypeptides (LMPs): LMP2, LMP7, and LMP10; (2) transporters associated with antigen processing (TAP): TAP1 and TAP2; (3) the chaperone proteins: calnexin, calreticulin, and tapasin; and (4) MHC class I heavy chain and β2-microglobulin [4–8]. Peptide antigens are transported into the endoplasmic reticulum (ER) by TAP and are loaded onto the MHC complex with the aid of the chaperone proteins. The functional MHC class I complexes are, in turn, transported to the cell surface and presented to T lymphocytes. Precursors of CTLs, through the T cell receptor, recognize foreign peptides derived from pathogens, and begin a cascade of activities leading to stimulation of specific immune responses against pathogen-infected cells. Overall, the expression of stable MHC class I molecules on the cell surface is regulated by the peptides supplied by TAP [1,9]. Antigen-presenting cells (APCs) derived from TAP1^−/−^ mice cannot transport antigenic peptides from the cytoplasm into the lumen of the ER for MHC class I binding and eventual presentation on the cell surface. Therefore, these cells lack the capacity to direct the priming of antigen-specific T cells [8].

The development of vaccine adjuvants that promote immunity at low doses of inocula is one approach to generate protection in individuals who would otherwise respond adversely to the administration of standard doses of inocula. Adverse responses to standard doses of inocula are a problem encountered in vaccination against viruses such as smallpox and, as a result, conventional vaccines cannot be administered to a significant fraction of the population who are either immune suppressed or who would otherwise react adversely to the established vaccine protocols [10]. As vaccination against a variety of pathogens becomes more widespread, there will be a greater need to increase the efficiency of the inocula while reducing the sizes of the batches of vaccine required for vaccination of an entire population. This would be particularly important during times of acute need, when rapid responses are required during an emergent epidemic. To increase vaccine potency and efficiency, a variety of adjuvants have been developed. These either suffer from substantial toxicity or cannot be implemented because their mode of action is obscure [11]. Here we report that a profound increase in T-cell-mediated immune responses to several infectious viruses was achieved using an immunization strategy involving a combination of both the infectious agents and a recombinant vaccinia virus (VV) containing a TAP-gene construct. This unexpected observation suggests that recombinant TAP may be used as a novel adjuvant to increase vaccine efficacy and potency.

## Results

### TAP Expression Is Required for Antigen-Specific H-2 (Mouse Major Histocompatibility Complex) K^b^ Surface Expression In Vitro

**Figure 1:**
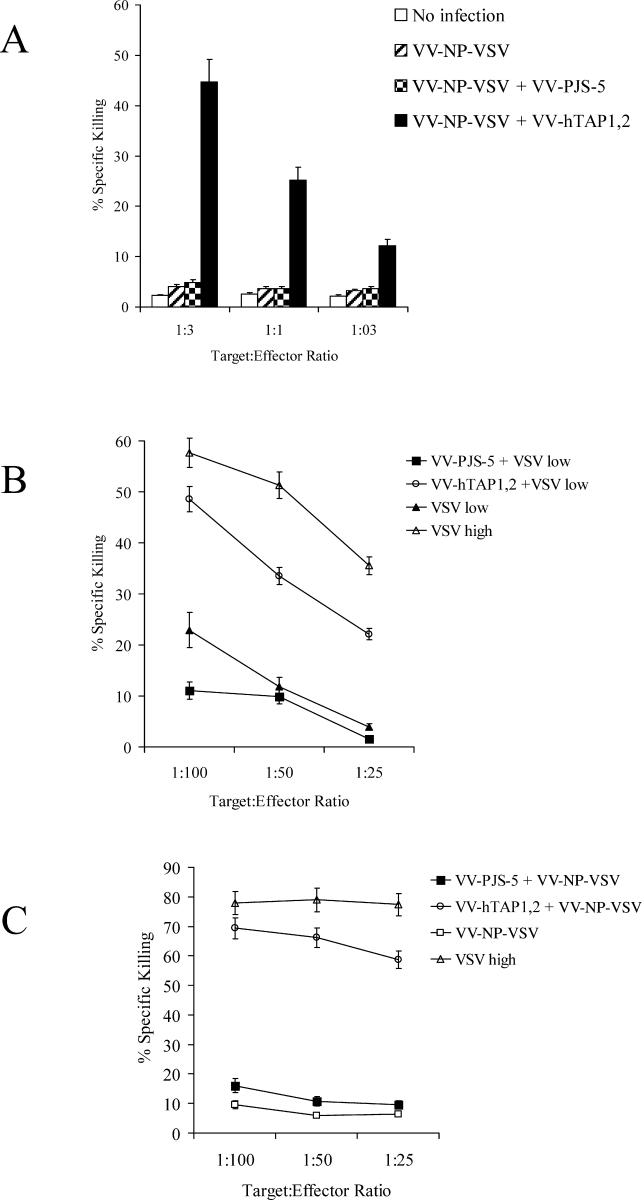
VV-hTAP1,2 Restores Antigen Processing in the TAP-Deficient Cell Line T2-K^b^ and Increases Immune Responses to VSV (A) A standard chromium-release assay was performed to establish the ability of VV-hTAP1,2 to restore antigen processing in the TAP-deficient cell line T2-K^b^. T2-K^b^ cells coinfected with VV-hTAP1,2 and VV-NP-VSV were used as targets, and splenocytes from VSV-infected mice were used as effectors. Targets coinfected with both VV-PJS-5 and VV-NP-VSV or infected with VV-NP-VSV alone, or uninfected cells, were used as negative controls for VV-hTAP1,2. (B) A standard chromium-release assay was performed to measure the ability of VV-hTAP1,2 to increase specific CTL activity in immunized mice. RMA cells pulsed with VSV-NP_55–59_ peptide were used as targets, and effectors were obtained from mice coinfected with VV-hTAP1,2 and low-dose VSV. Effectors from mice coinfected with VSV and VV-PJS-5 or a low dose of VSV alone were used as negative controls for the presence of VV-hTAP1,2 in the coinfections. Effectors from mice infected with a high dose of VSV demonstrated maximal CTL activity and were used as a positive control. (C) A standard chromium-release assay was used to confirm that the increase in immune responses was due to TAP-dependent transport of NP-VSV rather than to nonspecific effects of VV infection on antigen processing. RMA cells pulsed with VSV-NP_55–59_ peptide were used as targets, and effectors were obtained from mice coinfected with VV-hTAP1,2 and VV-NP-VSV. Effectors from mice infected with a high dose of VSV were used as positive controls for maximal CTL activity. Effectors from mice coinfected with VV-PJS-5 and VV-NP-VSV or from mice infected with VV-NP-VSV alone were negative controls for the presence of VV-hTAP1,2. Values represent the mean of triplicate measurements ± standard error of the mean.

T2-K^b^ cells express H-2K^b^ but lack both TAP1 and TAP2 [12,13] and have a very low expression of MHC class I on the cell surface owing to inefficient antigen processing. In a CTL assay, vesicular stomatitis virus (VSV)–specific effectors were able to lyse T2-K^b^ cells coinfected with VV containing minigene for VSV-NP_52–59_ (VV-NP-VSV) and recombinant vaccinia virus carrying human TAP1 and TAP2 (VV-hTAP1,2) in a dose-dependent manner. This indicated that a functional TAP complex was formed by the VV-hTAP1,2 infection, leading to high levels of H-2K^b^–vesicular stomatitis virus nucleoprotein (VSV-NP) surface expression (Figure 1A). In contrast, T2-K^b^ targets coinfected with VV-NP-VSV and VV encoding the plasmid PJS-5 (VV-PJS-5, negative control vector), or with VV-NP-VSV alone, were strongly resistant to lysis, and the level of lysis was similar to that in uninfected targets. These targets did not respond to increasing doses of effectors in the assay, indicating that the surface expression of H-2K^b^–VSV-NP_52–59_ is below the threshold for CTL recognition. The levels of lysis in the targets coinfected with VV-NP-VSV and VV-PJS-5, or with VV-NP-VSV alone are small (3%–5%) relative to the targets infected with VV-hTAP1,2 which are associated with up to 45% lysis.

### Increased TAP Expression Increases Immune Responses to VSV and Sendai Virus Infection In Vivo

Splenocytes from mice coinfected with the low dose of VSV and VV-hTAP1,2 showed dramatic increases in CTL activity against RMA cell targets (Figure 1B). The response was specific to the expression of TAP rather than to the VV vector alone, since coinfection with VV-PJS-5 did not increase CTL responses to VSV. To exclude the possibility that the effect seen with VV-hTAP1,2 was the result of VV-dependent alteration of proteasome function interacting with TAP over-expression, VV-NP-VSV was used in the coinfections as the source of antigen instead of VSV [14]. The epitope generated by VV-NP-VSV does not require degradation by the proteasome, and therefore any increase in CTL activity can be attributed to TAP over-expression. Mice coinfected with VV-NP-VSV and VV-hTAP1,2 exhibited dramatic increases in vaccinia virus carrying the vesicular stomatitis virus nucleocapsid protein minigene (VSV-NP_52–59_)–specific CTL responses when compared to coinfection with VV-NP-VSV and VV-PJS-5 (Figure 1C).

**Figure 2:**
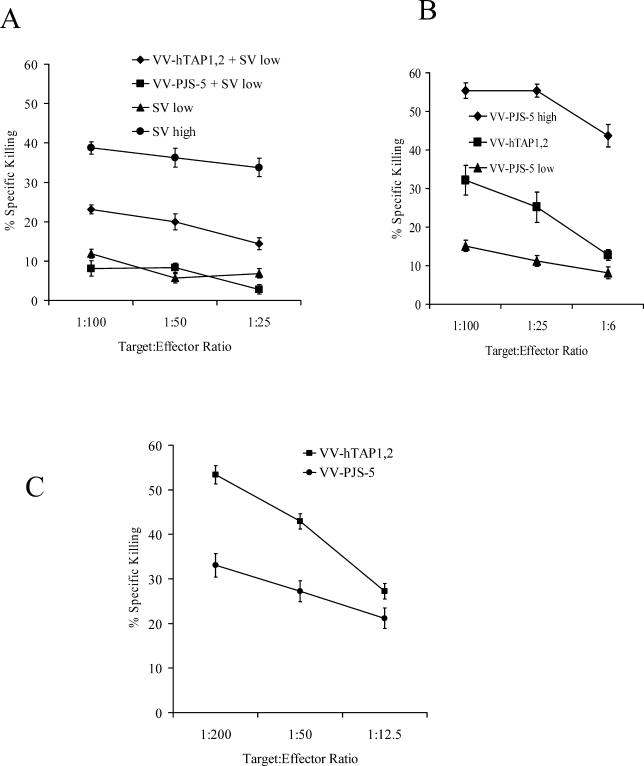
VV-hTAP1,2 Increases Antigen Presentation and Immune Responses to SV and VV in Mice (A) A standard chromium-release assay was used to determine the ability of VV-hTAP1,2 to increase immune responses to SV. RMA cells pulsed with SV-NP peptides were used as targets, and effectors were obtained from the mice coinfected with a low dose of SV and VV-hTAP1,2. The mice coinfected with a low dose of SV and VV-PJS-5 or with a low dose of SV alone were used as negative controls. Effectors from the mice infected with a high dose of SV were used as positive controls for maximal SV-specific CTL activity. (B) A standard chromium-release assay was used to determine the ability of VV-hTAP1,2 to stimulate VV-specific CTL responses. RMA cells infected with VV-PJS-5 were used as targets, and effectors were obtained from the mice vaccinated with a low dose of VV-hTAP1,2. Effectors from the mice vaccinated with an equivalent low dose of VV-PJS-5 were used as negative controls, and effectors from the mice vaccinated with a high dose of VV-PJS-5 were used as positive controls for maximal CTL activity. (C) A standard chromium-release assay was used to measure the ability of human TAP expression to increase antigen presentation in normal mouse splenocytes. Naïve splenocytes, which had been stimulated overnight with LPS (LPS blasts) and infected with VV-hTAP1,2, were used as targets for VV-specific effectors; VV-specific effectors were obtained from mice infected with VV-PJS-5. LPS blasts infected with VV-PJS-5 were used as negative controls. Values represent mean of triplicate measurements ± standard error of the mean.

Another well-characterized CTL epitope, from Sendai virus nucleoprotein (SV-NP), was investigated to provide further evidence that TAP over-expression can augment antiviral responses. The infectious dose of Sendai virus (SV) required to achieve minimal and maximal CTL responses was also first determined by titration (data not shown). As was the case for VSV, mice coinfected with SV and VV-hTAP1,2 exhibited an increased specific immune response against SV-NP epitope when compared to responses elicited by the vector controls (Figure 2A).

### Increased TAP Expression Increases Immune Responses to VV Infection In Vivo

The augmentation of a specific immune response against a virus by increasing TAP expression in APCs may require that viral infection and the over-expression of TAP occur in the same cell. To address this, we generated CTLs directed against antigen(s) derived from VV. VV-specific CTL activity in mice infected with a low dose of VV-hTAP1,2 was much greater than that from mice infected with an equivalent low dose of the control, VV-PJS-5 (Figure 2B).

### Increased TAP Expression Increases Endogenous Antigen Processing

We examined whether TAP over-expression could increase endogenous antigen processing. A VV-specific CTL assay was used to compare H-2K^b^ and H-2D^b^ VV-specific antigen processing in naïve splenocytes infected with VV-hTAP1,2 or VV-PJS-5. Naïve splenocyte targets infected with VV-hTAP1,2 were more susceptible to killing by VV-specific effectors than naïve splenocytes infected with VV-PJS-5 (Figure 2C).

### Increased TAP Expression Increases the Frequency of VSV-Specific CD8^+^

**Figure 3:**
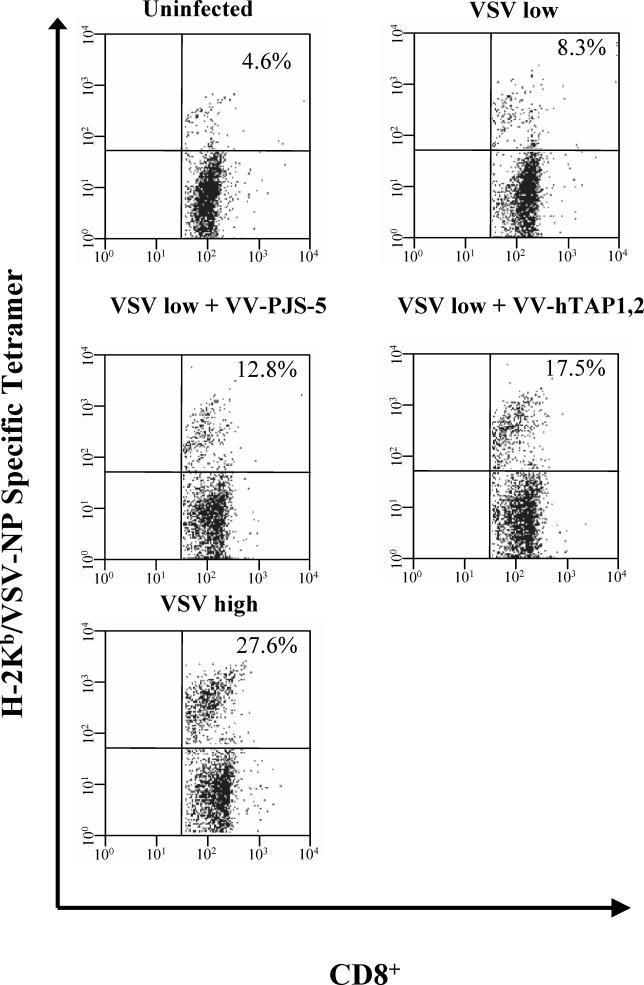
Antigen-Specific Tetramer Staining Was Used to Determine T-Cell Responses in Coinfections with VV-hTAP1,2 and VSV The percentage of CD8^+^ splenocytes specific for H-2K^b^–VSV-NP_52–59_ was determined by flow cytometry using double labeling with an anti-CD8^+^ antibody and a VSV-NP–specific tetramer. The value in the upper-right quadrant of the scatter-plots represents the percentage of CD8^+^ cells specific for H-2K^b^–VSV-NP_52–59_ for mice infected with a low dose of VSV and VV-hTAP1,2. The mice coinfected with both VSV and VV-PJS-5 or with a low dose of VSV, or uninfected mice, were used as negative controls for VV-hTAP1,2. The mice infected with a high dose of VSV alone were used as a positive control.

VSV-NP/K^b^-specific tetramer analysis compared the proportion of splenocytes specific for H-2K^b^ VSV-NP_52–59_ among CD8^+^ splenocytes between VSV-infected mice and mice coinfected with VV-hTAP1,2 and VSV (Figure 3). Mice coinfected with VV-hTAP1,2 and a low dose of VSV elicited a higher frequency of VSV-NP_52–59_–specific CD8^+^ splenocytes (17.5% of CD8^+^ splenocytes) than mice coinfected with VV-PJS-5 and low-dose VSV (12.8%), or with a low dose of VSV alone (8.3%). The differences between a combined VV-PJS-5 and VSV low-dose vaccination, on the one hand, and a combined VV-hTAP1,2 and VSV low-dose vaccination, on the other hand, were highly significant (z-statistic = 4.701, *p* < 0.0001). The maximum VSV-specific CD8^+^ frequency (27.6%) was observed with the highest dose of VSV infection.

### Peptide-Transport Activity and Human TAP Expression

**Figure 4:**
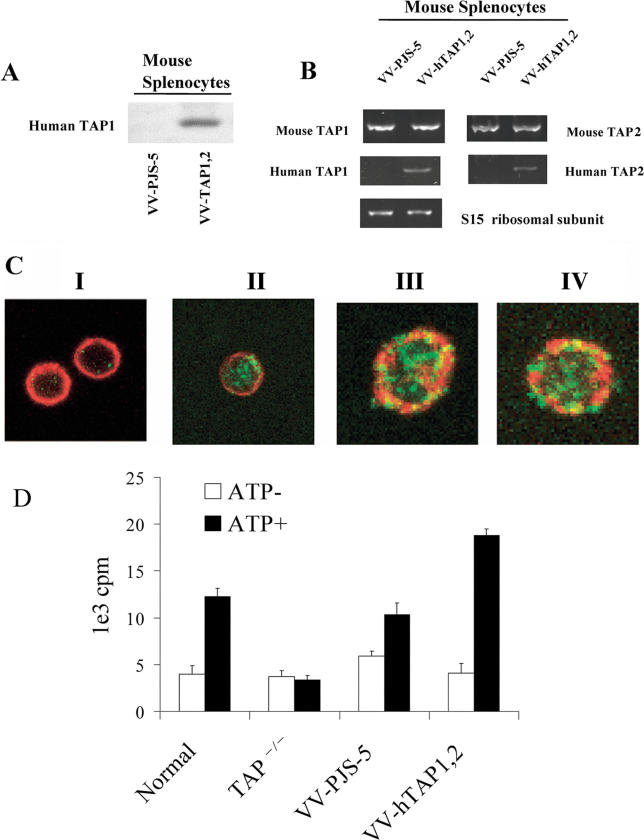
Human TAP Expression and Activity Was Determined in Splenocytes 24 h after the Mice Were Infected with VV-hTAP1,2 (A) Human TAP1 protein expression in mouse splenocytes was determined by Western blot. The mice infected with VV-PJS-5 were used as negative controls for human TAP1 expression. (B) The expression of human TAP1 and human TAP2 was detected by RT-PCR 24 h after the mice were infected with VV-hTAP1,2. The mice infected with VV-PJS-5 were negative for human TAP1 and TAP2. (C) Immunofluorescence visualized with confocal microscopy identified human TAP1 expression in antigen-presenting splenocytes isolated from mice 24 h after infection with VV-hTAP1,2. The mice infected with VV-PJS-5 were used as negative controls for human TAP1 expression (green fluorescence) (I). Cell-surface markers (red fluorescence) identified cell types. Representative images show the following cell types: (I) B cell from a mouse infected with VV-PJS-5 (negative control); (II) B cell that is positive for human TAP1; (III) macrophage that is positive for human TAP1; and (IV) DC that is positive for human TAP1. (D) ATP-dependent TAP activity was measured in splenocytes taken 24 h after the mice were infected with VV-hTAP1,2 or VV-PJS-5 (negative control). Active transport activity was measured in the presence or absence of ATP by a peptide-transport assay that determined the translocation of radioactive peptides from the cytosol into the ER. Normal uninfected mice, uninfected TAP^−/−^ mice, and mice infected with VV-PJS-5 were used as negative controls when assessing the effect of VV-hTAP1,2 infections on peptide-transport activity. The bars represent the mean value ± standard error of the mean of triplicate measurements. The data are representative of the experiment performed in duplicate.

Human TAP1 protein was detected in splenocytes by immunoblotting (Figure 4A), and RT-PCR analysis showed that both human TAP1 and human TAP2 mRNA were present (Figure 4B). Human TAP1 mRNA levels were quantified with real-time RT-PCR in splenocytes from mice 4, 8, and 24 h after intraperitoneal (i.p.) infection. Calculations based on the threshold cycle number indicated that the abundance of human TAP1 mRNA was equal to mouse TAP1 mRNA 4 and 8 h after infection, but decreased to 2% of endogenous mouse TAP1 mRNA by 24 h after infection. Immunocytochemistry showed that 7% of total splenocytes were positive for human TAP1. Double staining for cell-specific antigens and human TAP1 showed that 3.0% of B cells, 2.4% of macrophages, and 1.9% of dendritic cells (DCs) were positive for human TAP1 expression (Figure 4C). A peptide-transport assay clearly showed that splenocytes from VV-hTAP1,2–infected mice had a higher capacity to transport a 125I-labeled peptide library into the lumen of the ER than splenocytes from normal mice or from the mice infected with VV-PJS-5 (Figure 4D).

### Increased TAP Expression in DCs Increases MHC Class I–Restricted Presentation of Exogenous Antigens

**Figure 5:**
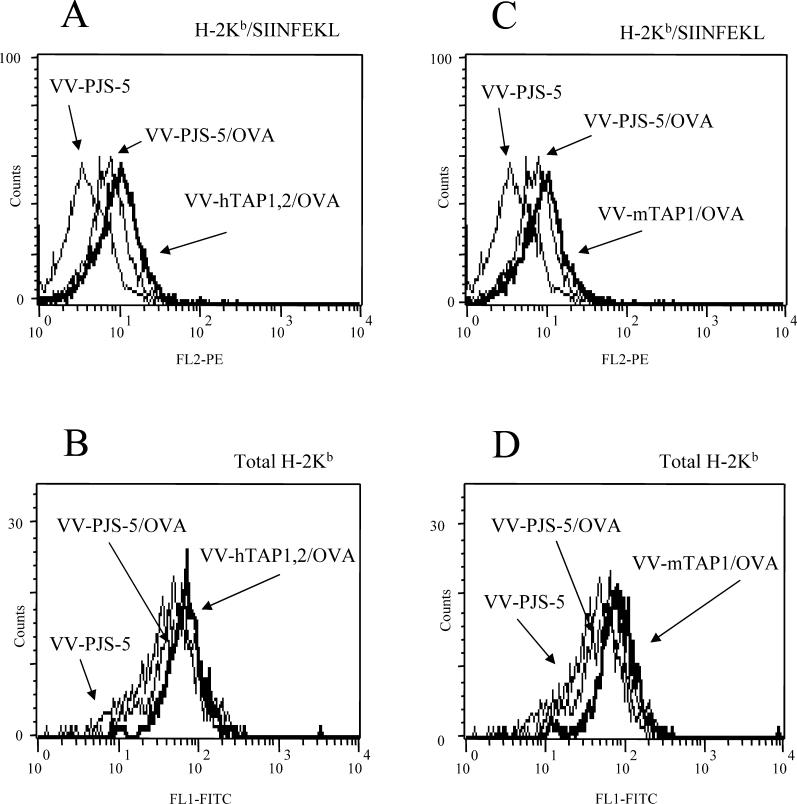
The Effect of VV-hTAP1,2 and VV-mTAP1 Infection on the Cross-Presentation Activity of OVA/SIINFEKL by Normal Spleen-Derived DCs DCs infected with VV-hTAP1,2 expressed greater (A) H-2K^b^–SIINFEKL and (B) total H-2K^b^ than DCs infected with VV-PJS-5. DCs infected with VV-mTAP1 also expressed greater (C) H-2K^b^–SIINFEKL and (D) total H-2K^b^ than DCs infected with VV-PJS-5. DCs infected with VV-PJS-5, but not incubated with OVA, served as negative controls for cross-presentation. The data are representative of the experiment performed in duplicate.

A subpopulation of splenocytes corresponding to the splenocyte DCs showed a 7.4% increase in total H-2K^b^ following infection with VV-hTAP1,2 when compared to V-PJS-5 (data not shown). This effect on DCs led us to investigate the cross-presentation of ovalbumin (OVA), an exogenously derived antigen. Treatment of DCs with VV-hTAP1,2 or recombinant vaccinia virus carrying mouse TAP1 (VV-mTAP1) significantly increased the amount of H-2K^b^–SIINFEKL expression when compared to DCs infected with VV-PJS-5 (*p* < 0.01). The assay was repeated three times, with each assay showing statistically significant increases in H-2K^b^–SIINFEKL and total H-2K^b^ expression by DCs infected with VV-hTAP1,2 or VV-mTAP1 (*p* < 0.01) (Figure 5A and 5B). DCs infected with VV-hTAP1,2 or VV-mTAP1 and incubated with OVA expressed significantly higher numbers of total H-2K^b^ complexes compared to VV-PJS-5–infected DCs (40% and 30% increase, respectively; *p* < 0.01) (Figure 5C and 5D).

### VV-hTAP1,2–Vaccinated Mice Resist Lethal Viral Challenge

**Figure 6:**
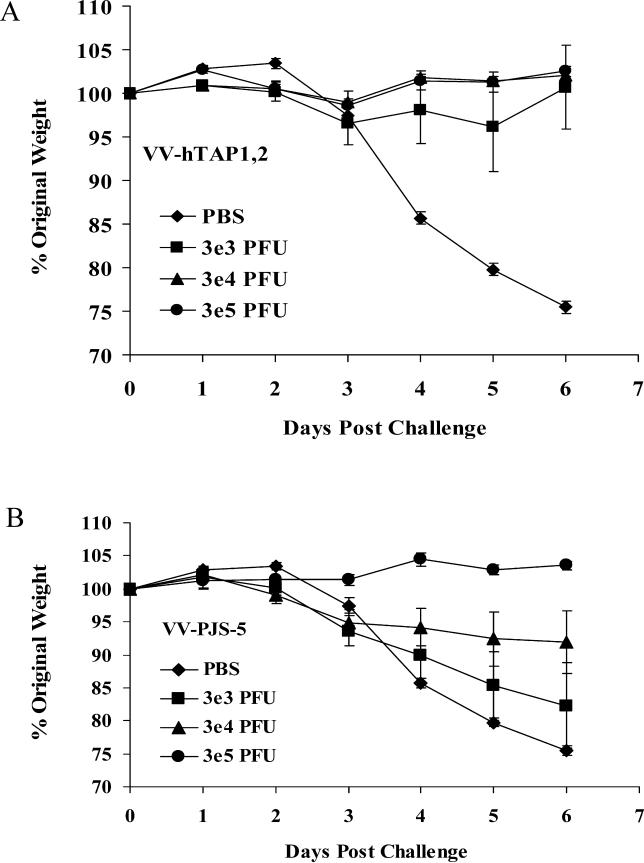
A Viral-Challenge Experiment Was Used to Measure the Protection Provided by Low-Dose Vaccination with VV-hTAP1,2 (A) Three groups of mice were vaccinated with escalating doses of VV-hTAP1,2 (3e3 PFU, 3e4 PFU, 3e5 PFU) and were challenged 14 d later with a lethal dose of VV-WR (1e5 PFU). Percentage weight change was measured as an indication of death and morbidity. Three doses of low-dose VV were administered. (B) Three Groups of mice were vaccinated with escalating doses of VV-PJS-5 (3e3 PFU, 3e4 PFU, 3e5 PFU), and were challenged 14 d later with a lethal dose of VV-WR (1e5 PFU). These groups served as negative controls for the effect of VV-hTAP1,2 on protection from lethal viral challenge. Mice vaccinated with PBS served as positive controls for lethal viral challenge. Data points represent mean weight changes ± standard error of the mean (*n* = 6) recorded daily.

A viral-challenge experiment determined whether TAP-dependent increases in immune function are significant in generating protective immune responses in vivo. Weight change was monitored in groups of mice, following vaccination with escalating doses of VV-hTAP1,2, VV-PJS-5, or PBS, and then administration of a lethal vaccinia virus Western Reserve strain (VV-WR) challenge [15–18]. Five out of six mice receiving the lowest VV-hTAP1,2 vaccine doses survived the challenge with minimal weight loss (less than 5%) and returned to normal, pre-challenge weight within 6 d. All the mice vaccinated with the intermediate and high doses of VV-hTAP1,2 survived challenge without weight loss. In contrast, the mice vaccinated with the lowest dose of VV-PJS-5 suffered significant morbidity (approximately 20% weight loss) and high mortality (four out of six mice died). The mice vaccinated with the intermediate dose of VV-PJS-5 also experienced high morbidity and one death. These mice were unable to regain weight to the pre-challenge level until 14 d after viral challenge. Mice receiving the highest dose of VV-PJS-5 were completely protected. All sham-vaccinated mice (PBS) were dead by 7 d post-challenge, consistent with a 1e5 plaque-forming unit (PFU) intranasal challenge (Figure 6A and 6B). VV-hTAP1,2 vaccination provided significantly greater protection than vaccination with VV-PJS-5 (*p* < 0.05) in a dose-dependent manner (*p* < 0.01). Mice vaccinated with VV-hTAP1,2 at the lowest doses were able to resist a lethal challenge equivalent to the highest vaccine dose of VV-PJS-5. This represents a 100-fold increase in the efficacy of VV-hTAP1,2 to generate a protective immune response compared with VV-PJS-5.

## Discussion

Contrary to expectation, we observed that increasing TAP expression in mice augments the cellular immune response to a variety of viral pathogens including VSV, SV, and VV at infectious doses that normally do not generate significant CTL activity. These responses do not appear to be the result of a reversal of viral immuno-evasion mechanisms with respect to antigen presentation since VSV and SV are not known to down-regulate MHC class I surface expression. The correlation of immune responses with increasing levels of viral infection in mice reflects the fact that CTL priming requires a threshold level of relevant viral peptides to be expressed on the surface of APCs [19–22]. The increase in CTL activity in mice coinfected with VV-hTAP1,2 and with low infectious doses of VSV, SV, and VV-NP-VSV shows that this increase is dependent on the expression of TAP activity alone.

It is unlikely that this augmentation is due to the more efficient translocation of VSV-derived peptides by human TAP and/or interspecies (human/mouse) TAP heterodimers compared to mouse TAP complexes. It has been reported that human TAP preferentially transports peptides containing either hydrophobic or positively charged amino-acids at their C-terminus, while mouse TAP is slightly more restrictive and favors peptides with hydrophobic amino-acids at their C-terminus [23]. The VSV-NP and SV-NP are two murine K^b^-restricted epitopes that contain the same hydrophobic residue (leucine) at the C-terminus. The transport of these peptides by human TAP would compete with an additional peptide pool containing positively charged C-terminal residues. This might lead to a reduced amount of VSV-NP or SV-NP entering the ER lumen for surface presentation through human TAP heterodimers.

In addition, the SV-NP epitope has an aromatic residue (phenylalanine) at the peptide position 1 (N-terminus), and this has a strong deleterious effect for human TAP binding [24] and for transport. Therefore, we conclude that the transport of SV-NP by human TAP would be no better than by mouse TAP. For interspecies TAPs, the transport of peptides is restricted to those with hydrophobic C-terminal residues, similar to mouse TAP [25]. This would imply that when mouse TAP1 or TAP2 associates with its human TAP counterpart, they play a dominant role in selecting the peptides for transport. Furthermore, once the transport-permissive peptide, for example SV-NP, binds to interspecies TAPs, the phenylalanine residue at the N-terminus that is in contact with the human TAP counterpart may limit its binding capacity and, therefore, its transport. For these reasons, we conclude that the augmentation of the CTL responses against viruses in our experiments is justified by TAP over-expression rather than by the increased efficiencies of interspecies TAP heterodimers.

The priming of T cells requires cell-to-cell contact, and therefore APCs adjacent to T cells play a critical role [2,9]. The increase in VSV-specific CD8^+^ cells observed with VV-hTAP1,2 coinfections indicates a TAP-dependent increase in APC cross-priming and cross-presentation activity and is explained by an increase in TAP expression and peptide-transport activity in the APCs of the spleen. To reconcile the increase in peptide-transport activity observed in the translocation assays with the frequency of human TAP-positive splenocytes, we estimate that the human TAP-positive splenocytes need to express 12 to 17 times more human TAP1 mRNA than endogenous mouse TAP1. This was confirmed by the high expression of the human TAP gene early in the infection.

DCs generate virus-specific CTLs via cross-presentation of exogenously acquired viral antigens in the context of MHC class I molecules [26]. This is achieved by a TAP-dependent process although additional TAP-independent pathways have been described recently [27–29]. Increased expression of mouse TAP1 appeared to be as effective as increased expression of human TAP1 and TAP2 in raising the level of MHC class I complexes (H-2K^b^–SIINFEKL) on the cell surface. Expression of TAP1 alone has been shown to stabilize TAP2 protein and TAP2 mRNA in TAP-deficient cells and could explain the effectiveness of TAP1 alone [30]. We conclude that supra-normal expression of TAP increases endogenous antigen processing (see Figure 2C) and the levels of both total and cross-presented MHC class I antigens on the surface of DCs, thereby leading to greater CTL responses in vivo.

Under normal conditions, it is estimated that only one-third of all TAP molecules translocate peptides actively. During an acute viral infection, however, TAP activity increases significantly owing to the rapid increase in the available intracellular peptide pool [31]. Therefore, the supply of peptides to MHC class I molecules is theoretically the limiting factor in antigen presentation. It is known that increasing the delivery of peptides into the ER by the artificial creation of a signal-sequence peptide fusion increases antigen presentation and immune responses [32]. However, these experiments bypass TAP altogether, and the relative amounts of peptide generated in the two pathways are difficult to equilibrate and, therefore, to compare. Our results indicate that during a viral infection there is competition between self and viral peptides in the MHC class I binding-peptide pool, limiting the amount of viral epitopes reaching the lumen of the ER and subsequently the cell surface. Increased TAP activity leads to more viral epitopes presented on the cell surface by MHC class I, resulting in increased immune responses. In DCs, over-expression of TAP has the additional effect of increasing the cross-presentation pathway for MHC class I, a pathway believed to be unique to these cells. A framework for how cross-presentation may operate has been published recently [26,27,33,34]. Endocytosed or phagocytosed exogenous antigens gain access to one or more types of vesicles where loading of antigenic peptide onto nascent MHC class I molecules is thought to occur. MHC class I molecules gain access to the endolysosomal compartment by virtue of a tyrosine-based sorting signal in their cytoplasmic domains [26]. It has been suggested that TAP molecules reside in this compartment and that a fusion event with the ER may make antigen processing more efficient for both exogenous and endogenous antigens. Over-expression of TAPs in the endogenous and the exogenous antigen-processing compartments of DCs could increase the efficiency of both pathways, leading to enhanced specific immune responses without stimulation of detectable autoimmune responses (D. Waterfield, unpublished data).

The presence of TAP genes in VV provides protection against lethal viral challenges at 100-fold lower amounts of inocula than VV without TAP genes. The inclusion of TAP in vaccination regimens acts as a gene-based adjuvant to boost immune responses against viral antigens, thereby allowing for reduced vaccine doses. Increased immune responses in response to low-dose vaccinations are desirable in the elderly and the very young, in whom immune systems may be compromised [35]. An additional advantage of including TAP as an adjuvant is its ability to increase peptide transport of a number of immunogenic peptides simultaneously, thereby aiding in the delivery of diverse peptides for binding to most HLA (human major histocompatibility complex) class I alleles expressed in the immunized population. TAP could be used as an adjuvant in peptide vaccines, but its use does not have to be restricted to viral vectors. For example, it could also be injected in other forms, such as in DNA plasmids attached to gold particles, or in any other system that inserts the TAP complex directly into the cell's protein-processing pathway [36]. Finally, the use of TAP as an adjuvant has the advantage that we have a solid intellectual understanding of its mechanism of action. This appears to be lacking in the case of many other generalized adjuvants [37]. We have shown here, in an animal model, that TAP over-expression can augment cell-mediated immunity against the cowpox virus (vaccinia), a close relative of smallpox (variola). It is conceivable that this approach could have applications in augmenting responses against smallpox in humans.

Recently, clinical trials for some promising HIV-vaccine candidates have been suspended because of poor cellular immune responses [38]. Inclusion of TAP in such vaccines may be able to improve their efficacy. Future clinical experiments will help to further establish whether the inclusion of TAP in vaccine regimens has advantages over existing protocols and whether other components of the intracellular antigen-processing pathway(s) are also limiting in healthy individuals. Nonetheless, the approach that has been discovered is novel and may have tremendous potential for vaccination of humans and animals against a variety of infectious diseases.

## Materials and Methods

### Animals, cells, and viruses.

The mouse strain C57BL/6 (H-2K^b^) was obtained from Jackson Laboratories (Bar Harbor, Maine, United States) and housed at the Biotechnology Breeding Facility (University of British Columbia) according to the guidelines of the Canadian Council on Animal Care. Mice (5–12 wk of age) were screened for pathogens with the Murine ImmunoComb Test (Charles River Laboratories, Wilmington, Massachusetts, United States).

VSV, Indiana Strain (a gift from Frank Tufaro, University of British Columbia) was cultured on vero cells (American Type Culture Collection [ATCC], Manassas, Virginia, United States). VV-hTAP1,2 and VV-mTAP1 (both gifts from John Yewdell, NIH/NIAID, Bethesda, Maryland, United States), VSV-NP_52–59_, VV-NP-VSV, VV-PJS-5 (PJS-5 a gift from Bernard Moss, NIH/NIAID), and VV Western Reserve strain (a gift from Shirley Gillam, University of British Columbia) were cultured on CV-1 cells [5,39,40]. SV was purchased from ATCC. CV-1 cells were cultured in DMEM/10% FBS and vero cells, and RMA cells were cultured in RPMI/10% FBS. VSV and VV infectious units were determined by tissue-culture infectious-dose assay (TCID50) or by standard plaque assay. SV infectious units were determined by chicken-egg infectious-dose (CEID50) as indicated on the label. T2 cells negative for TAP1 and TAP2 and transfected with mouse H-2K^b^ were a gift from Peter Cresswell, (Yale University, New Haven, Connecticut, United States).

### Generation of VSV, SV, and VV-specific effectors.

H-2K^b^–restricted virus-specific CTLs were generated by i.p. injection of mice with VSV (low-dose [3.6e4 TCID50] or high-dose [1.5e7 TCID50]), SV (low-dose [1.6e5 CEID50] or high-dose [1.6e7 CEID50]), VV-PJS-5 (low-dose [3e4 PFU] or high-dose [5e6 PFU]), VV-hTAP1,2 (3e4 PFU), and VV-NP-VSV (3e6 PFU). Titrations determined the infectious doses of VSV and SV that would generate a minimum (low dose) and maximum (high dose) CTL response against peptide-pulsed RMA targets. Splenocytes were harvested 6 d after infection and cultured (RPMI-1640/10% FBS complete media) for 2–3 d to eliminate nonspecific natural killer cell killing. For VSV infection alone or via coinfection with VV-hTAP1,2 or VV-PJS-5, splenocytes were incubated without peptide. For infections with SV or VV-NP-VSV, splenocytes were stimulated with SV peptide (1 μM SV-NP_324–332_ peptide, FAPGNYPAL) or VSV-NP peptide (1 μM VSV-NP_52–59_ peptide, RGYVYQGL). VV-hTAP1,2 or VV-PJS-5–infected mouse splenocytes were restimulated in vitro for 3 d with irradiated syngeneic splenocytes infected with VV-PJS-5 (3.4e7 PFU/1e8 syngeneic splenocytes).

### Cytotoxicity assays.

Cytotoxic activity was measured in standard 4-h 51Cr-release assays using T2-K^b^ cells, RMA cells, or naïve splenocytes as targets. T2-K^b^ targets were infected with VV-NP-VSV alone or in combination with VV-hTAP1,2 or VV-PJS-5 (multiplicity of infection [MOI] = 10) for 6 h. The RMA target cells were pulsed with VSV-NP_52–59_ peptide (5–25 μM) or SV-NP_324–332_ peptide (5–25 μM) for the relevant CTL assay. For VV antigen-specific killing, the RMA targets were infected overnight with VV-PJS-5 (MOI = 0.34). For the measurement of endogenous antigen processing, targets were generated by the in vitro stimulation of naïve splenocytes (2e7 cells) for 2 d with lipopolysaccharide (LPS) (Escherichia coli J5 LPS [1 μg/ml], Calbiochem, San Diego, California, United States), followed by overnight infection with either VV-PJS-5 or VV-hTAP1,2 (5e6 PFU).

### Detection of CD8^+^/VSV-NP_52–59_–specific H-2K^b^–restricted splenocytes.

The frequency of CD8^+^/VSV-NP_52–59_–specific splenocytes was measured in two separate experiments using a H-2K^b^–VSV-NP_52–59_ epitope-specific tetramer conjugated to phycoerythrin (Custom iTAg MHC Tetramer, Beckman Coulter Canada, Mississauga, Ontario, Canada) and a fluorescein-conjugated rat anti-mouse CD8a (Ly-2) monoclonal antibody (clone 53–6.7) after Fc receptor block (clone 2.4G2, Mouse Fc Block), (BD Biosciences Pharmingen, San Diego, California, United States). Mice were coinfected with VV-PJS-5 (3e4 PFU) plus low-dose VSV, or VV-hTAP1,2 (3e4 PFU) plus low-dose VSV, or infected with low-dose VSV, or high-dose VSV. Six days after infection, CD8*^+^*/VSV-NP_52–59_–specific fluorescence as a percentage of total CD8^+^ fluorescence was determined by flow cytometry.

### Detection of human TAP expression in splenocytes.

Human TAP1 expression in splenocytes from the mice infected with VV-hTAP1,2 was determined by SDS-PAGE and Western blot. The blots were probed for human TAP1 with rabbit anti-human TAP1 antiserum (Stressgen Biotechnologies, Victoria, British Colombia, Canada) and visualized by enhanced chemiluminescence (Amersham Biosciences, Little Chalfont, United Kingdom).

### Quantitative RT-PCR for human and mouse TAP1 in splenocytes.

RT-PCR was used to detect human TAP1 and TAP2, and mouse TAP1 and TAP2, in spleens 24 h after infection. In addition, a time-course quantification of human TAP1 and mouse TAP1 was performed using quantitative real-time PCR (QRT-PCR). Total RNA was extracted (RNeasy Midi Kit, Qiagen, Valencia, California, United States) from mouse spleens 4 h, 8 h, and 24 h after i.p. infection with VV-hTAP1,2 (3e4 PFU). QRT-PCR reactions (Sigma-Genosys Canada, Oakville, Ontario, Canada) were performed in duplicate using a Light Cycler (Roche Diagnostics, Mannheim, Germany) for mouse TAP1 and for human TAP1, plus a ribosomal small subunit S15. The sequences of the primer pairs used in the RT-PCR and the QRT-PCR are listed in Table 1.

**Table 1:**
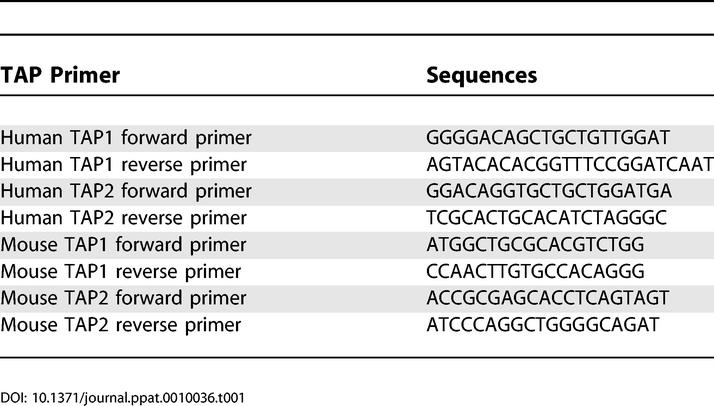
Sequences of the Primer Pairs Used in the RT-PCR and the QRT-PCR

The threshold cycle (CT) above background was determined for mouse TAP1 and human TAP1 and normalized to the lowest S15 CT value among the reactions. The differences in CT were used to calculate the abundance of human TAP1 relative to mouse TAP1. Relative abundance = 2 (CTmTAP1–CThTAP1). The CT values represent the average of three mice per time point.

### Visualization of human TAP expression in splenocytes.

Visualization of human TAP expression in spleen-derived antigen presentation cells from mice infected with VV-hTAP1,2 or VV-PJS-5 was performed using confocal fluorescence microscopy. Splenocytes were double labeled with rabbit anti-human TAP1 antiserum (Stressgen Biotechnologies) and one of the following cell-surface markers: rat anti-mouse B220 (B cell marker, BD Biosciences Pharmingen), rat anti-mouse MAC-1 (macrophage marker, BD Biosciences Pharmingen), or rat anti-mouse NLDC-145 antibodies (DC marker) (gift from Ralph Steinman, the Rockefeller University, New York, New York, United States). The presence of human TAP1 was determined in approximately 300 cells per surface marker in 20 randomly chosen fields. Splenocytes from VV-PJS-5–infected mice were used as negative controls.

### Transport activity of human TAP in mouse splenocytes.

TAP heterodimer activity, in the presence or absence of adenosine triphosphate (ATP), was detected by a streptolysin-O–mediated peptide-transport assays in splenocytes harvested 24 h after mice were infected with VV-hTAP1,2 or VV-PJS-5 using a radio-iodinated peptide library containing a glycosylation site (NXT) (125I, specific activity, 10 Ci/mmol) [41,42]. Splenocytes from uninfected normal mice and TAP^−/−^ mice were used as controls.

### Cross-presentation of OVA peptides (SIINFEKL) by DCs.

Splenic DCs were isolated using CD11c+ magnetic beads run through an AutoMacs (Miltenyi Biotec, Auburn, California, United States) after enrichment by centrifugation (Ficoll Paque Plus, Amersham Biosciences). DCs were infected with VV-PJS-5, VV-mTAP1, or VV-hTAP1,2 (3e6 cells/1e4 PFU) for 2 h, followed by incubation with GM-CSF (10 ng/ml complete RPMI 1640) and OVA (10 mg/ml) (Worthington Biochemical, Lakewood, New Jersey, United States) or PBS (18 h at 37 °C). DCs stained with phycoerythrin–conjugated rat anti-mouse CD11c antibody, Fc blocker (Fcγ III/II receptor), and either FITC-conjugated anti-H-2K^b^ antibody (clone AF6–88.5) (BD Biosciences Pharmingen) or 25.D1.16 antibody specific for K^b^/SIINFEKL (a gift from R. Germain, NIH/NIAID) [43]. Flow cytometry was used to quantify H-2K^b^ and H-2K^b^–SIINFEKL complexes.

### VV-WR–challenge experiments.

VV-hTAP1,2 and VV-PJS-5 viruses were demonstrated to replicate equally in cell culture. Forty-two mice were randomized into seven groups (six mice per cage) and were vaccinated with three different doses of VV-hTAP1,2 or VV-PJS-5 (3e3, 3e4, and 3e5 PFU in 300 μl PBS i.p.) or PBS. Fourteen days later, mice were weighed and challenged with a lethal dose of VV-WR (1e5 PFU in 20 μl of clarified cell lysate delivered intranasally) under isoflurane anesthesia. Weight was recorded daily for 14 d, and any mouse falling below 25% of pre-challenge weight was euthanized. Mean weight going forward was calculated from the remaining survivors.

### Statistical analysis.

The z-statistic was calculated to determine the statistical significance of the differences in the proportions of H-2K^b^, VSV-NP, and CD8+ cells generated by the vaccination protocols. A two-way ANOVA, after square-root transformation of the data, was used to analyze the main effects of dose and the recombinant VV on mouse weight 5 d after VV-WR challenge. Bonferroni procedure corrected *p*-values for multiple comparisons. A chi-square test (univariate comparison, using FlowJo 3.7.1 [Treestar, http://www.treestar.com]) compared flow-cytometry histograms for differences in total H-2K^b^ or H-2K^b^–SIINFEKL complexes. *p*-Value of < 0.01 (99% confidence interval) was considered significant, and T(X) > 10 was empirically determined as a cut-off value.

## Supporting Information

### Accession Numbers

The Swiss-Prot (http://www.ebi.ac.uk/swissprot) accession numbers for the proteins discussed in this paper are calnexin (BC040244), calreticulin (NM 007591), LMP2 (U22919), LMP7 (U22031), LMP10 (U77784), MHC class I heavy chain (V00747) and β2-microglobulin (NM 009735), TAP1 (NM 013683), TAP2 (NM 011530), and tapasin (A7316613). The GenBank (http://www.ncbi.nlm.nih.gov/Genbank) accession numbers for the strains discussed in this paper are mouse strain C57BL/6 (V00746), recombinant VV carrying human TAP1 (NM 000593) and TAP2 (NM 018833) genes, SV (X00087), VSV, Indiana Strain (J02428), and VV Western Reserve strain (AY243312).

## Abbreviations

APC: antigen-presenting cell
ATP: adenosine triphosphate
CEID50: chicken-egg infectious-dose
CT: threshold cycle
CTL: cytotoxic lymphocyte
DC: dendritic cell
ER: endoplasmic reticulum
H-2: mouse major histocompatibility complex
HLA: human major histocompatibility complex
i.p.: intraperitoneal
LMP: low molecular-weight polypeptide
LPS: lipopolysaccharide
MHC: major histocompatibility complex
MOI: multiplicity of infection
OVA: ovalbumin
PFU: plaque-forming unit
QRT-PCR: quantitative real-time PCR
SV: Sendai virus
SV-NP: Sendai virus nucleoprotein
TAP: transporter associated with antigen processing
TCID50: tissue-culture infectious-dose assay
VSV: vesicular stomatitis virus
VSV-NP: vesicular stomatitis virus nucleoprotein
VSV-NP_52–59_: vesicular stomatitis virus nucleocapsid protein minigene
VV: vaccinia virus
VV-hTAP1,2: recombinant vaccinia virus carrying human TAP1 and TAP2
VV-mTAP1: recombinant vaccinia virus carrying mouse TAP1
VV-NP-VSV: vaccinia virus containing minigene for VSV-NP_55–59_
VV-PJS-5: vaccinia virus encoding the plasmid PJS-5
VV-WR: vaccinia virus Western Reserve strain
